# Navigating the Usher Syndrome Genetic Landscape: An Evaluation of the Associations between Specific Genes and Quality Categories of Cochlear Implant Outcomes

**DOI:** 10.3390/audiolres14020023

**Published:** 2024-02-26

**Authors:** Micol Busi, Alessandro Castiglione

**Affiliations:** Department of Audiology, Orebro University Hospital, Interdisciplinary Research in Clinical Audiology—IRCA, Orebro University, 70116 Orebro, Sweden; alessandro.castiglione@regionorebrolan.se

**Keywords:** Usher syndrome, sensorineural hearing loss, retinitis pigmentosa, cochlear implantation, genetic mutations, therapeutic approaches, gene therapy

## Abstract

Usher syndrome (US) is a clinically and genetically heterogeneous disorder that involves three main features: sensorineural hearing loss, retinitis pigmentosa (RP), and vestibular impairment. With a prevalence of 4–17/100,000, it is the most common cause of deaf-blindness worldwide. Genetic research has provided crucial insights into the complexity of US. Among nine confirmed causative genes, *MYO7A* and *USH2A* are major players in US types 1 and 2, respectively, whereas *CRLN1* is the sole confirmed gene associated with type 3. Variants in these genes also contribute to isolated forms of hearing loss and RP, indicating intersecting molecular pathways. While hearing loss can be adequately managed with hearing aids or cochlear implants (CIs), approved RP treatment modalities are lacking. Gene replacement and editing, antisense oligonucleotides, and small-molecule drugs hold promise for halting RP progression and restoring vision, enhancing patients’ quality of life. Massively parallel sequencing has identified gene variants (e.g., in *PCDH15*) that influence CI results. Accordingly, preoperative genetic examination appears valuable for predicting CI success. To explore genetic mutations in CI recipients and establish correlations between implant outcomes and involved genes, we comprehensively reviewed the literature to gather data covering a broad spectrum of CI outcomes across all known US-causative genes. Implant outcomes were categorized as excellent or very good, good, poor or fair, and very poor. Our review of 95 cochlear-implant patients with US, along with their CI outcomes, revealed the importance of presurgical genetic testing to elucidate potential challenges and provide tailored counseling to improve auditory outcomes. The multifaceted nature of US demands a comprehensive understanding and innovative interventions. Genetic insights drive therapeutic advancements, offering potential remedies for the retinal component of US. The synergy between genetics and therapeutics holds promise for individuals with US and may enhance their sensory experiences through customized interventions.

## 1. Introduction

Usher syndrome (US) is a complex and heterogeneous genetic disorder that impairs both hearing and vision. It is the most inherited form of deaf-blindness and is characterized by the combination of sensorineural hearing loss (SNHL) and retinitis pigmentosa (RP) [1,2]. With a global prevalence of 4–17/100,000, US is a leading contributor to cases of deaf-blindness worldwide [3,4,5,6,7]. These debilitating conditions, arising from genetic mutations, converge to create a unique clinical landscape [8]. In addition to auditory and visual impairments, certain subtypes of US involve vestibular dysfunction, impacting the sense of balance in the affected individuals [9,10,11,12]. Therefore, the syndrome has been clinically subdivided into three main clinical types, USH1, USH2, and USH3, based on the severity and age at onset of symptoms [3,12]. USH1 is characterized by profound congenital SNHL, RP, and vestibular dysfunction, and USH2 involves moderate to severe congenital SNHL and RP. Conversely, USH3 is associated with post-lingual SNHL and RP [7]. The comprehensive spectrum of symptoms profoundly impacts a person’s communication ability, mobility, and overall quality of life [13,14,15].

US is characterized by substantial genetic complexity. Genetic advancements have facilitated the identification of US-causative genes. *MYO7A*, *USH1C*, *CDH23*, *PCDH15*, and *USH1G* (*SANS*) are associated with USH1, *USH2A*, *ADGRV1*, and *WHRN* are involved in USH2, and *CLRN1* is the sole confirmed gene associated USH3 [7]. Different types of US are associated with the presence of several mutations in different genes, leading to specific subclinical presentations and subtypes. For example, subtypes USH1B, USH1C, and USH1D are caused by mutations in *MYO7A*, *USH1C*, and *CDH23*, respectively. The genetic landscape of US is further complicated by the existence of digenic, bi-allelic, and polygenic forms, as well as the potential for genetic variants to manifest as non-syndromic RP and deafness [7]. Recent research has illuminated the involvement of additional genes in atypical phenotypes, further underscoring the genetic complexity of US [3].

The discovery of US-causative genes has enhanced our understanding of the molecular mechanisms driving US, as well as has paved the way for precision diagnostics and targeted therapies. Further, research findings have underscored the potential to enhance the quality of life in affected individuals. Progress is being made in therapeutic approaches for US. Cochlear implants (CIs) and hearing aids have been pivotal in addressing hearing loss and have significantly improved auditory and communication capabilities in individuals with US [3]. Particularly, CIs have revolutionized the management of SNHL and enhanced patients’ quality of life [6,16]. However, the challenge remains to address retinal degeneration associated with RP, for which effective remedies are not available to date and which significantly affects a person’s communication abilities and spatial orientation. Gene therapy and emerging drug therapies hold promise for the treatment of retinal degeneration associated with RP [5,12]. Gene therapies, gene editing, and precision medicine offer hope for halting symptom progression, as well as for partially or fully restoring impaired senses [6]. While there remain challenges, such as validating gene causality and understanding rare mutations, the pursuit of a comprehensive understanding of causative genes paves the way to more effective treatments and improved outcomes for patients with US. Genotype–phenotype correlations have been established for selected US-related genes, aiding in prognosis and treatment strategies [7].

Early diagnosis, facilitated by these advancements, is increasingly being recognized as a pivotal step toward effective interventions that can significantly impact disease management and patient outcomes. Genetics combined with early interventions presents a promising avenue for enhancing the quality of life of individuals with US. However, given the complex interplay between genetic factors, clinical interventions, and prognostic outcomes, there are multifaceted considerations. In the context of US, early diagnosis holds dual significance: it enables timely interventions that can help mitigate the progression of SNHL and RP. These early interventions include a range of strategies, from cochlear implantation to gene therapies, that can lead to substantial improvements in communication abilities and visual function.

The synergy between next-generation sequencing, early diagnosis, and interventions offers great promise for individuals with US. However, the incorporation of genetic prognostic factors into clinical decision-making demands a nuanced understanding of the interplay between genetics and clinical realities. While genetics can guide interventions, clinical outcomes are the ultimate benchmark. Balancing these aspects ensures that early interventions are not only guided by genetic insights but are tailored to each individual’s unique clinical needs, allowing the most effective management of US.

To improve CI outcomes, genetics studies have been conducted to elucidate potential correlations between gene mutations and post-implantation results. However, while the identification of genetic determinants is useful, recognizing the multifaceted nature of CI outcomes is crucial. In the exploration of whether mutations significantly influence implant outcomes, it is essential to acknowledge that genetics is only one facet of the broader landscape.

To address the gaps in the literature, we aimed to shed light on the complex relationship between genetics and CI outcomes. We identified genes that positively (“favorable” genes) and negatively (“unfavorable” genes) affect CI outcomes through a comprehensive literature review study of all cases published as of 2000.

## 2. Materials and Methods

### 2.1. Literature Review

A comprehensive literature review was conducted to identify the relevant studies exploring genetic effects on CI outcomes in patients with US. PubMed, Embase, and other relevant databases were searched using keywords such as “CI outcomes”, “genetic factors”, and “Usher”. Articles published between 2000 and the present were included to ensure that recent insights were captured. Studies focusing on the relationship between specific genetic variants and CI outcomes were prioritized. The study inclusion criteria were as follows: (1) studies involving CI recipients with known genetic mutations, (2) studies reporting outcomes in terms of speech perception, auditory performance, or quality of life, and (3) studies providing detailed genetic information, including gene variants and associated functional effects.

Extracted data included patient demographics, genetic mutations, implant outcomes, and follow-up duration. Pearson’s chi-squared test and odds ratios (ORs) were used to explore associations between specific genetic variants and CI success rates. Two expert otologists with demonstrated experience in genetics, hearing loss, US, and CIs independently, randomly, and blindly classified cochlear outcomes into four arbitrary categories: 75–100% of available test reach was defined as a very good or excellent outcome; 50–74% was considered a good outcome; 25–49% was defined as a fair or poor outcome; <25% was regarded a very poor or clearly poor outcome. We acknowledge that this is a very arbitrary and speculative assessment of outcomes, which should ideally also consider pre-operative assessment and various other important factors, including diagnosis and outcome predictions. However, a validated, standardized classification to evaluate the effectiveness of hearing rehabilitation is currently not available.

The collective of studies included in this review had the following limitations: (1) limited availability of long-term follow-up data, (2) heterogeneity in study designs and outcome measures, (3) variability in genetic testing methodologies and criteria for CI success, (4) potential confounding factors such as age, duration of hearing loss, and pre-implantation auditory function.

The results were interpreted considering the complex nature of CI outcomes, acknowledging the multifaceted contributions of genetic, physiological, anatomical, and cognitive factors.

### 2.2. Statistical Analysis

Statistical analysis for investigating potential associations between genetic mutations and CI outcomes was performed using Pearson’s chi-squared tests. A gene–outcome matrix was constructed to discern patterns and trends in the genetic influences on CI success. Odds Ratios (ORs) and their associated *p*-values were calculated to examine the association between specific genes and CI outcomes. An aggregated analysis was conducted for outcomes classified as “Positive” (combining “Excellent” and “Good” categories) versus “Less Positive” (combining “Fair or Poor” and “Very Poor” categories). Genes with unavailable data (“NA”) were excluded from the analysis. If necessary, Yates’ continuity correction was applied to prevent computational errors and infinite odds ratios. The statistical significance of each OR was assessed using Fisher’s exact test, suitable for the small sample sizes in some categories. Statistical analysis was performed using R software (version 4.3.2).

### 2.3. Categorical Evaluation

Category 1, very good or excellent: significant improvement in speech perception (75–100% correct on speech tests), high satisfaction reported, notable functional gain, and CAP categories 6–7. CAP (Categories of Auditory Performance) categories are a scale used to assess auditory performance in individuals who have received a cochlear implant. This scale ranges from 0 to 7, where each category represents a higher level of auditory skill, starting from no awareness of environmental sounds (0) to the ability to use a telephone with a familiar speaker (7). CAP categories help in evaluating the benefits of cochlear implants in terms of understanding speech and sounds, providing a standardized way to measure auditory improvements post-implantation.

Category 2, good: noticeable improvement in speech perception (50–74% correct), satisfactory patient reports, and stable CAP from 4 to 5 measures.

Category 3, fair or poor: limited improvement in speech perception (25–49% correct), some dissatisfaction reported by the patient, or concerns on CAP categories not more than 2–3.

Category 4, very poor or far below expectations: minimal to no improvement in speech perception (<25% correct), no substantial difference with hearing aids, significant dissatisfaction, or concerning measures indicating 0–1 CAP categories, similar to potential device issues.

It is important to note that outcomes can vary widely based on various factors, including age at implantation, duration of deafness, cause of hearing loss, rehabilitation and training, and the device’s technology. Therefore, these categorizations are general and may require adjustments based on individual circumstances.

## 3. Results

### 3.1. Genetic Landscape of Usher Syndrome Patients with Cochlear Implants

Mutations in the nine causative genes of Usher Syndrome (US) have been identified in 95 patients who underwent cochlear implantation surgery, with outcomes available for statistical analysis for the purposes of this study. (Table 1): *MYO7A*, *USH1C*, *CDH23*, *PCDH15*, and *USH1G* (*SANS*) for USH1; *USH2A*, *ADGRV1*, and *WHRN* for USH2 and *CLRN1* for USH3 (Table 1). Variants in *MYO7A*, *USH1C*, *CDH23*, *PCDH15*, and *WHRN* are also responsible for non-syndromic hearing loss associated with loci DFNB2 and DFNA11, DFNB18, DFNB12, DFNB23, and DFNB31, respectively. The remaining genes are not involved in other forms of hearing loss to the best of our knowledge [17].

### 3.2. Genetic Factors and CI Outcomes

The Pearson chi-squared test statistic was 28.64, with a *p*-value of 0.0179 and a statistical power for α = 0.05 of 99.97%, indicating a statistically significant difference in outcomes across the different genes, with 15 degrees of freedom.

The *p*-values obtained from the Pearson Chi-square test for each gene, comparing the outcome categories “Excellent”, “Good”, “Fair/Poor”, and “Very poor” within each gene, are as follows: *MYO7A*: *p* = 0.91; *CDH23*: *p* = 0.41; *PCDH15*: *p* = 0.0008; *USH1G*: *p* = 0.22; *USH2A*: *p* = 0.0001 and *CLRN1*: *p* = 0.36.

These *p*-values represent the probability of observing the differences in the frequencies of the outcome categories within each gene.

From the results, it can be discerned that *PCDH15* and *USH2A* exhibit statistically significant differences in their outcome distributions (*p* < 0.05). The other genes (*MYO7A*, *CDH23*, *USH1G*, *CLRN1*) do not show statistically significant differences in their outcome distributions (*p* ≥ 0.05).

The overall OR for an “excellent (>75%)” over a “very poor (<25%)” outcome was 3.18, with a *p*-value of 0.0027, suggesting a significant association between genetic makeup and outcome. Table 2 shows different ORs for each gene and relative category. The ORs above 1 for *MYO7A*, *CDH23*, and *CLRN1* indicate a higher probability of achieving an “excellent” outcome over a “poor” or “very poor” outcome. Conversely, the OR below 1 for *PCDH15* indicates a lower likelihood of an “excellent” or “good” outcome. The *p*-values provide insight into the statistical significance of the associations, with only variants in *USH2A* showing a statistically significant association with “excellent” outcomes. The significant OR for *PCDH15* indicates that patients with mutations in this gene are significantly less likely to have an “excellent” outcome than patients carrying mutations in the other genes. Conversely, the significant OR for *USH2A* suggests that patients with mutations in this gene are significantly more likely to have an “excellent” outcome, as no “poor” or “very poor” outcomes were reported for this gene.

The aggregate analysis revealed varying degrees of association between the examined genes and the likelihood of “Positive” or “Less Positive” outcomes, as summarized in Table 3. Notably, the gene USH2A exhibited a markedly high OR, suggesting a strong association with “Positive” outcomes, whereas *PCDH15* showed a minimal OR, indicating a lesser likelihood of “Positive” outcomes associated with this gene. The *p*-values from Fisher’s exact test provided insight into the statistical significance of these associations. For instance, the association for *USH2A* was found to be statistically significant (*p* < 0.05), underscoring a potential link between this gene and favorable outcomes. Conversely, other genes like *MYO7A*, *CDH23*, and *CLRN1*, despite having ORs greater than 1, did not reach statistical significance.

These findings suggest a differential impact of specific genes on the aggregated outcomes, with some genes showing a stronger association with “Positive” outcomes than others. The statistical analysis, particularly the use of Fisher’s exact test, provided a robust framework for assessing the significance of these associations, taking into account the small sample sizes in some categories.

### 3.3. Specific Gene Mutations and CI Outcomes

The literature search for *USH1C*, *ADGRV1*, and *WHRN* mutations and CI outcomes did not yield articles explicitly discussing the relationship between such mutations and CI outcomes. Theriot et al. aimed to determine the success of CIs in patients with *USH1C* mutations through a retrospective chart review [38]. They evaluated demographic data, genetic diagnosis, CI devices, and post-implantation hearing and audio-verbal therapy. Their findings revealed that 28 out of 109 participants (25.7%) had CIs, and the USH1C subtype was genetically confirmed. At present, patients with *USH1C* mutations typically receive CIs. However, the data available in [38] were not useful for statistical analysis in the present study. Imtiaz et al. reported a novel truncating mutation in *USH1G* (p.S243X) that is associated with the disease phenotype. They also discussed novel retinal findings and CI outcomes in individuals affected by this mutation, suggesting that CIs have been successfully used in patients with this *USH1G* mutation [24]. Yoon et al. reported successful CI outcomes in two patients with mutations in *CDH23* and *ADGRV1*. Both patients showed a clear improvement in ton audiometry, from 120 and 70 dBHL (median pre-implant) to 25 and 22.5 dBnHL (median post-implant), respectively [33]. However, speech audiometry data were not available for the purposes of this review study.

Genes for which data were not available or not useful for statistical analysis were excluded from the analysis.

## 4. Discussion

Our findings indicate that mutations in specific genes significantly influence the outcomes of CIs in patients with US, emphasizing the need for a personalized approach to treatment selection [39,40,41,42,43]. Although not all results demonstrate statistically significant values, certain genes appear more likely to be associated with “positive” outcomes, as opposed to less favorable or “negative” outcomes, following cochlear implantation. It is evident that the results may be influenced by various biases, and larger sample sizes are required to confirm these hypotheses. However, the statistical significance for mutations in two genes, *USH2A* and *PCDH15*, not only supports pre-existing hypotheses but also aligns with both the pre- and post-synaptic lesion theory [43], as well as the spiral ganglion theory [42]. The *USH2A* gene, which is almost exclusively expressed in the inner ear and more broadly in the peripheral organ, contrasts with the *PCDH15* gene. *PCDH15* is widely expressed throughout the central nervous system and plays a crucial role in the development of synapses in the auditory pathway. Mutations in *PCDH15* can thus significantly impact auditory processing (https://www.proteinatlas.org/ accessed on 18 February 2024).

Nevertheless, the pivotal question of whether gene mutations influence CI outcomes necessitates a comprehensive view. Cochlear implantation is a complex procedure influenced by variables ranging from an individual’s overall health and age to cochlear anatomical characteristics and surgical techniques. Therefore, genetic information is only a fragment of the broader picture. It is important to acknowledge that genetics, while potentially influential, is not the sole determinant of CI outcomes.

Assuming, despite the study limitations and as suggested by previous research [39,40,41,42,43], that the success of cochlear implantation significantly varies depending on the genotype, genetic testing could become an integral part of the preoperative evaluation for CI candidates. It may help predict the likelihood of successful auditory outcomes post-implantation and assist in personalized treatment planning, optimizing the timing of intervention, and tailoring post-operative rehabilitation strategies to the individual’s genetic profile. However, the identification of a gene potentially favorable for treatment outcomes should not be construed as a guaranteed predictor of success.

Understanding why the success of cochlear implantation significantly varies due to *PCDH15,* or why *USH2A* may favor functional outcomes post-implantation is complex. Although all reported CI cases demonstrated improved performance when compared with rehabilitation using hearing aids alone, the results were not always entirely satisfactory.

Despite the potential implications, mutations in certain genes should not be viewed as contraindications to surgery, according to our current knowledge. While genetic mutations can be useful for counseling, management, or rehabilitation planning, they should not be regarded as an absolute barrier to cochlear implantation. The use of genetic information as a prognostic factor is constrained by numerous variables, including age at implantation, the extent of hearing loss, and the onset of symptoms [44].

The variability in CI outcomes due to genetic factors is a subject of considerable research interest. In this perspective, CI outcomes are determined by the molecular etiology of deafness. However, the examination of individual genes involved in US reveals a complexity that is difficult to reconcile with the varied conditions and exceptions observed. Most of the genes under examination are expressed not only in the cochlea but also in the brain. This prompts inquiry into why the effects of mutations would be circumvented in both the cochlea and the brain. Additionally, assuming this hypothesis was accurate, we would expect hearing aid outcomes to be similar yet converse to those observed with CIs. However, this is not evident, as outcomes with hearing aids often significantly differ from those with CIs [3,7,13,14,15,43,44,45,46].

Davies et al. [29] conducted a systematic review and found that cochlear implantation improved sound detection, speech perception, speech intelligibility, and quality of life in most patients with US. Jatana et al. [16] reported that children with US who received CIs achieved significant open-set speech perception and oral communication skills. Hoshino et al. found that late implantation in US patients allowed for sound detection, but speech recognition was more successful in patients with previous hearing stimulation [45]. Nair et al. compared CI outcomes between children with US and a control group and found that speech and language acquisition improved but to a lesser extent than in the normative cohort [46]. Overall, these findings suggest that cochlear implantation can provide hearing benefits for individuals with US. However, the outcomes vary depending on factors such as age at implantation and previous hearing stimulation. Evaluating gene expression in the central nervous system does not provide definitive evidence about the link between genetic mutations and adverse outcomes with CIs.

Advancements in the field highlight the potential to improve the lives of those affected by US. Elucidating the genetic mosaic of US informs therapeutic strategies, with gene therapy and new pharmacological approaches showing promise for treating retinal degeneration associated with RP [5,12]. Innovations in gene therapies, gene editing, and precision medicine offer promise for not only halting but also potentially reversing the progression of symptoms and restoring lost senses [7].

## 5. Conclusions

The debate surrounding the practical application of genetic prognostic factors in interventions for US is not unwarranted. While genetics can offer valuable insights, clinical outcomes remain central. Cochlear implantation, particularly when guided by genetic information, continues to provide superior outcomes compared to traditional hearing aids, reinforcing the importance of individualized patient care. Speculations of potential correlations between genetic profiles and post-implantation outcomes do exist. However, they must be critically examined within the context of complex genetic interactions and multifactorial determinants of clinical response.

Additional research is essential to determine the specific roles of various genes in US and to understand how they influence CI outcomes. The present literature review study provides valuable insights for personalized treatment approaches and underscores the need for further research to elucidate the complexities of genetic influences on CI success.

## Figures and Tables

**Table 1:** Outcomes associated with nine US-related genes. *NA* = Data not available or not useful for statistical analysis.

		Categories					
		1	2	3	4		
Gene	US Type	Excellent (>75%)	Good (50–75%)	Fair or Poor (25–50%)	Very Poor (<25%)	Total for Each Gene	References
*MYO7A*	USH1	6	5	3	2	16	[16,17,18]
*USH1C*	USH1	NA	NA	NA	NA	NA	
*CDH23*	USH1	8	13	6	5	32	[18,19,20,21]
*PCDH15*	USH1	2	1	5	4	12	[19,22,23]
*USH1G*	USH1	2	1	0	0	3	[24]
*USH2A*	USH2	10	3	0	0	13	[25]
*ADGRV1*	USH2	NA	NA	NA	NA	NA	
*WHRN*	USH2	NA	NA	NA	NA	NA	
*CLRN1*	USH3	4	8	6	1	19	[26]
Total in each category		32	31	20	12	95	[16,17,18,19,20,21,22,23,24,25,26,27,28,29,30,31,32,33,34,35,36,37]

**Table 2:** Odds Ratio (OR), 95% confidence interval in square brackets, and *p*-value for each gene and category. NS = not significant; NA = Data not available or not useful for statistical analysis; * *p* < 0.01.

Gene	1-Excellent OR [95% conf. int.]	2-Good OR [95% conf. int.]	3-Fair or Poor OR [95% conf. int.]	4-Very Poor OR [95% conf. int.]
*MYO7A*	1.25 [0.41–3.81]	0.97 [0.30–3.07]	0.93 [0.24–3.63]	1.14 [0.23–5.79]
*CDH23*	0.56 [0.22–1.44]	1.70 [0.70–4.16]	0.84 [0.29–2.44]	1.51 [0.44–5.19]
*PCDH15*	0.42 [0.09–2.03]	0.23 [0.03–1.86]	3.24 [0.90–11.62]	4.70 [1.15–19.15] *
*USH1G*	3.42 [0.30–39.17]	1.23 [0.11–14.10]	NA	NA
*USH2A*	8.07 [2.03–32.05] *	0.64 [0.16, 2.50]	NA	NA
*CLRN1*	0.49 [0.15, 1.64]	1.68 [0.60, 4.73]	2.08 [0.67, 6.41]	0.46 [0.06, 3.82]

**Table 3:** Association between USH-related genes and positive clinical outcomes. The table summarizes the corrected Odds Ratios (ORs) and *p*-values, calculated using Fisher’s Exact Test with Yates’ continuity correction, to assess the likelihood of “positive outcomes” (combining ‘Excellent’ and ‘Good’ categories) relative to “less positive outcomes” (combining ‘Fair/Poor’ and ‘Very Poor’ categories) for each gene. ORs greater than 1 suggest a higher likelihood of positive outcomes associated with the gene, while *p*-values below 0.05 indicate statistical significance.

Gene	OR	*p*-Value
*MYO7A*	2.48	0.303
*CDH23*	1.82	0.321
*PCDH15*	0.012	<0.001
*USH1G*	21.00	0.143
*USH2A*	261.00	<0.001
*CLRN1*	2.16	0.343

## Data Availability

Data are contained within the article.
